# Systematic review of cure and recurrence rates following minimally invasive parathyroidectomy

**DOI:** 10.1002/bjs5.77

**Published:** 2018-05-28

**Authors:** H. Ishii, R. Mihai, J. C. Watkinson, D. S. Kim

**Affiliations:** ^1^ Department of Ear, Nose and Throat, Head and Neck Surgery St George's Hospital London UK; ^2^ Department of Surgery Great Ormond Street Hospital London UK; ^3^ BUPA Cromwell Hospital London UK; ^4^ Department of Endocrine Surgery John Radcliffe Hospital Oxford UK

## Abstract

**Background:**

The majority of patients with primary hyperparathyroidism (PHPT) have a single overactive adenoma. Advances in preoperative imaging and surgical adjuncts have given rise to minimally invasive parathyroidectomy (MIP), with lower complication rates in comparison with bilateral neck exploration. Misdiagnosis and undertreatment of multiglandular disease, leading to potentially higher recurrence rates, remains a concern. This study evaluated risks of long‐term (1 year or more) recurrence following ‘targeted’ MIP in PHPT.

**Methods:**

Multiple databases were searched for studies published between January 2004 and March 2017, looking at long‐term outcomes (1 year or more) following targeted MIP for PHPT. English‐language studies, with at least 50 patients and a mean follow‐up of 1 year, were included.

**Results:**

A total of 5282 patients from 14 studies were included. Overall mean recurrence and cure rates were 1·6 (range 0–3·5) and 96·9 (95·5–100) per cent respectively. Mean follow‐up was 33·5 (1–145) months. When intraoperative parathyroid hormone (PTH) measurements were not done, cure rates were higher (99·3 per cent versus 98·1 per cent with use of intraoperative PTH measurement; P < 0·001) and recurrence rates lower (0·2 versus 1·5 per cent respectively; P < 0·001).

**Conclusion:**

Targeted MIP for a presumed single overactive adenoma was associated with very low recurrence rates, without the need for intraoperative PTH measurement when preoperative imaging studies were concordant. Targeted MIP should be encouraged.

## Introduction

Primary hyperparathyroidism (PHPT) is a common condition, with an estimated incidence of one to seven per 1000 adults^1^. The condition is detected incidentally in more than 80 per cent of subjects on routine biochemical analysis^2^ ^3^. Surgery remains the only curative option.

Parathyroid surgery via an open bilateral four‐gland neck exploration was first performed in 1925^4^ and remained the standard treatment until the early part of the 21st century. In experienced hands, this method has a cure rate of at least 95 per cent, with a morbidity rate of less than 3 per cent^5^, and does not require any form of preoperative localization imaging.

It is widely recognized that in over 85 per cent of patients with PHPT the cause is a single overactive parathyroid adenoma, often identifiable through preoperative imaging, allowing selective removal^5^. The two most common modalities of preoperative localization are sestamibi imaging and high‐resolution ultrasonography. A meta‐analysis^6^ examining the value of ultrasound imaging in PHPT found an overall pooled sensitivity of 76·1 per cent and a positive predictive value of 93·2 per cent, although operator and centre variation was acknowledged^7^. With negative localization of a solitary adenoma, the likelihood of multiglandular disease is reported to be up to 30 per cent^8^ ^9^.

Surgical management of PHPT has evolved over the past 20 years. Rapid intraoperative parathyroid hormone (IOPTH) assay^10^, sestamibi scintigraphy and radio‐guided parathyroidectomy^11^ have emerged, facilitating the development of ‘targeted’ parathyroidectomy techniques based on single‐gland excision via unilateral neck exploration. Various techniques have been described^12^, ^13^, ^14^, ^15^, with a general consensus that parathyroidectomy is classified as ‘minimally invasive’ when performed with preoperative localization through an incision of less than 2·5–3 cm^5^ ^12^, ^13^.

Targeted minimally invasive parathyroidectomy (MIP) has been shown in large studies, systematic reviews and meta‐analysis to be highly effective with low complication rates compared with open bilateral neck exploration (BNE)^14^, ^15^, ^16^. Advantages of MIP include shorter duration of surgery, lower rates of postoperative hypocalcaemia, less postoperative pain and a smaller scar^17^. There are also potential financial advantages in performing MIP^18^ ^19^, with lower operative costs and more rapid hospital discharge.

The consensus statement published by the European Society of Endocrine Surgeons (ESES)^20^ described MIP as a safe and cost‐effective procedure for the treatment of selected patients with PHPT. In the UK, the National Institute for Health and Care Excellence (NICE) released guidelines for minimally invasive video‐assisted parathyroidectomy (MIVAP)^21^, stating that ‘current evidence on the efficacy and safety of MIVAP is adequate to support the use of this procedure’.

Misdiagnosis and the risk of undertreating multigland disease, leading to high recurrence rates, nevertheless remains a potential shortcoming of this approach. This systematic review aimed to examine long‐term (1 year or more) recurrence rates following targeted MIP in PHPT.

## Methods

### Acquisition of evidence

The PRISMA protocol^22^ was followed to perform a comprehensive literature search using MEDLINE, Embase, CINAHL, the UK Clinical Trials Gateway and the US Trials Database between January 2004 and March 2017. The PICO framework^23^ was used and terms combined with Boolean operators (AND, OR) to refine the search further.

Two independent reviewers identified all studies that met the inclusion criteria for full review. References of the searched studies were evaluated for potential inclusion in the review. Where possible, contact was attempted with authors to verify data that were not clearly described or to confirm that specific data were not available from the study. The study included all articles published between January 2004 and March 2017 that reported MIP alone or comparison with BNE, involving at least 50 patients, where recurrence rates were documented and mean follow‐up was at least 1 year. Parathyroidectomy was considered minimally invasive if the authors documented incision of 3 cm or less, or if MIP was clearly stated. Only articles published in English were considered, and no age limits were set.

To analyse the best available data focusing on long‐term (follow‐up of at least 1 year) recurrence and cure rates, the inclusion criterion for centres performing MIP regularly (at least 50 patients per annum) was set. This was done to minimize skewing of data from ‘low‐volume’ centres and small case series.

### Study quality and levels of evidence

The quality of studies and risk of bias were assessed by two reviewers. All studies included in the review were non‐randomized, and therefore the Methodological Index for Non‐Randomized Studies (MINORS) tool^24^ was used. This tool assessed non‐randomized studies on the following criteria: clearly stated aims, inclusion of consecutive patients, prospective data collection, appropriate endpoints, unbiased evaluation of endpoints, appropriate duration of follow‐up and loss to follow‐up no more than 5 per cent. For comparative studies, further criteria were assessed: whether the control group underwent a standard intervention, the use of contemporary groups, baseline equivalence of the groups, prospective calculation of the sample size and statistical analysis adapted to the study design.

To ascertain the level of evidence of the included studies, the Oxford Centre for Evidence‐based Medicine (OCEM)^25^ guidelines were employed.

### Outcomes

The primary outcome was recurrence rate, defined as the rate of hypercalcaemia occurring after 6 months of normocalcaemia following parathyroidectomy.

Secondary outcomes were: cure rate (defined as normocalcaemia persisting for more than 6 months after surgery), type of MIP performed, use of IOPTH measurements and postoperative complication rate.

When analysing IOPTH use and non‐use during MIP, the studies were divided into those that included only patients who exhibited positive preoperative concordant imaging (PC cohort) and those that included a heterogeneous cohort (H cohort: 1–2 image‐positive or image‐negative patients).

### Statistical analysis

GraphPad Prism^®^ version 7.0d (GraphPad Software, La Jolla, California, USA) was used for statistical analysis, and Microsoft Excel^®^ version 16.12 (Microsoft, Redmond, Washington, USA) for data handling. The statistical significance of categorical variables was determined with Fisher's exact test. *P* < 0·050 was considered statistically significant.

## Results

### Study identification

**Figure 1:**
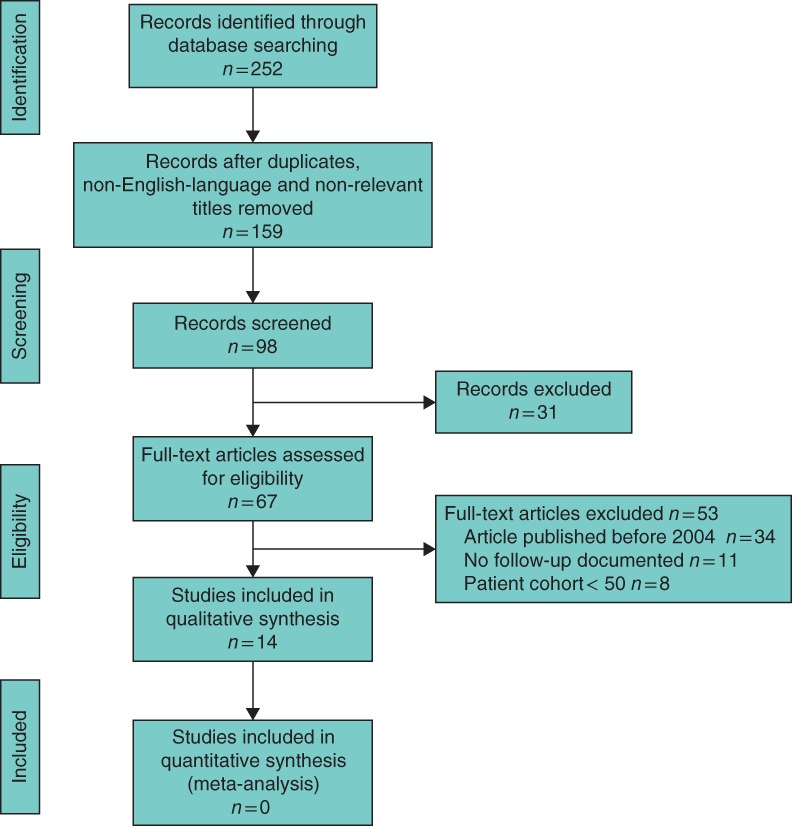
PRISMA diagram for the study

The initial literature search yielded a total of 252 studies, of which 14^11^ ^20^, ^26^, ^27^, ^28^, ^29^, ^30^, ^31^, ^32^, ^33^, ^34^, ^35^, ^36^, ^37^ met the inclusion criteria (*Fig*. 1). All studies were observational and included a total of 5282 patients who had targeted MIP for PHPT. There was a female preponderance of 3·4 : 1 and the overall mean age was 58·9 years (*Table* 1).

**Table 1:** Summary of included studies

Reference	Review period	Study type	No. of patients	Sex ratio (M : F)	Age (years)*	Mean recurrence rate (%)*	Mean cure rate (%)*	Follow‐up (months)*
Chen *et al*.^26^	1990–2004	Retrospective	188	n.d.	60(3)	1·1	99·0	48
Cohen *et al*.^27^	1999–2004	Retrospective	139	32 : 107	60(15) (16‐88)	0·0	98·6	15(12) (3–38)
Barczynski *et al*.^20^	2000–2006	Prospective	115	18 : 97	57·1(12·2)	0·9	98·3	28(10) (6–42)
Mihai *et al*.^32^	2001–2006	Prospective	150	46 : 104	60(16) (17–89)	0·0	99·3	15(10) (3–48)
Fouquet *et al*.^33^	2001–2008	Prospective	200	35 : 165	63·1 (13–87)	0·5	98·0	13(13) (6–72)
Venkat *et al*.^34^	2004–2009	Prospective	200	51 : 149	58·7(11·9) (13–88)	2·5	97·0	37(19) (6–72)
Barczyński *et al*.^28^	2003–2012	Retrospective	455	57 : 398	54·7(15·6) (18–82)	2·3	99·6	43(16) (12–112)
Chow *et al*.^29^	2002–2012	Retrospective	105	29 : 76	61·5(14·6)	0·0	n.d.	56·9
Norlén *et al*.^31^	1990–2013	Retrospective	2531	593 : 1938	61·8(13·9)	1·8	95·5	78 (1–24)†
Day *et al*.^35^	2003–2013	Prospective	556	147 : 409	61·1(13·4)	2·0	97·3	44 (1–145)
Yang *et al*.^30^	2008–2012	Retrospective	115	37 : 78	46 (28–57)†	0·9	99·1	24
Nijhuis *et al*.^37^	2000–2013	n.d.	114	n.d.	n.d.	3·5	96·5	34†
Thier *et al*.^36^	1989–2010	Prospective	292	58 : 234	66† (i.q.r. 57–75)	0·3	1 year: 98·5	60 (1–180)†
							5 years: 99·4	
							10 and 15 years: 100	
Kim *et al*.^11^	2000–2012	Retrospective	122	28 : 94	50·8(13)	0·0	99·2	45·5(42·2)

* Values are mean(s.d.) (range) unless indicated otherwise;

† values are median (range) unless indicated otherwise. n.d., Not documented.

### Quality of studies and levels of evidence

Of the 14 studies, seven were retrospective^11^ ^26^, ^27^, ^28^, ^29^, ^30^, ^31^, six involved collection of prospectively identified items^20^ ^32^, ^33^, ^34^, ^35^, ^36^ and one^37^ did not clarify the type of study. The median MINORS score was 10 of 16 (mean 9·8, range 6–14) for all studies, and the median of the four comparative studies^11^ ^20^, ^26^ ^28^ was 15 of 24 (mean 14·25, range 10–17).

According to the OCEM criteria, all of the included studies were classified as individual cohort studies and were therefore classified as having level 2b evidence.

### Outcomes

The overall mean recurrence rate for the 5282 patients was 1·6 (range 0–3·5) per cent, and the overall cure rate 96·9 (95·5–100) per cent. The overall mean duration of follow‐up for the studies included was 33·5 (1–145) months (*Table* 1).

All studies defined cure as normalization of serum calcium levels, and all recorded serum calcium levels at each follow‐up appointment. All but three studies^26^ ^31^, ^37^ also measured postoperative serum PTH levels routinely.

All studies used the widely accepted definition of ‘disease recurrence’ as hypercalcaemia after 6 months of proven normocalcaemia following initial surgery.

The most common type of surgery performed was the open MIP technique^26^ ^27^, ^29^, ^30^, ^31^, ^32^ ^34^, ^35^, ^36^, ^37^, but other approaches included MIVAP^20^ ^28^, minimally invasive radio‐guided parathyroidectomy^11^ and a totally endoscopic MIP technique^33^. When reported, studies had incision lengths of less than 3 cm, whereas studies that did not report on incision length stated that their procedure was ‘minimally invasive’ or a ‘focused/lateral exploration’.

Four studies^11^ ^29^, ^31^ ^32^ with a total of 2908 patients performed targeted MIP without IOPTH measurements, and nine studies^20^ ^26^, ^27^, ^28^ ^30^, ^33^, ^34^, ^35^, ^36^ carried out targeted MIP with IOPTH measurements in 2072 of the patients. One study^37^ made no reference to the use of this test, so was not included in the analysis. Five studies^28^, ^29^, ^30^, ^31^, ^32^ included only patients with positive, concordant preoperative localization studies on at least two different imaging modalities, seven^11^ ^20^, ^27^ ^33^, ^34^, ^35^, ^36^ included patients regardless of imaging findings, and two^26^ ^37^ did not report on imaging findings.

When IOPTH measurement was not done during MIP, overall mean cure rates were higher (99·3 per cent *versus* 98·1 per cent when IOPTH measurement was done; *P* < 0·001) and recurrence rates were lower (0·2 *versus* 1·5 per cent respectively; *P* < 0·001) (*Table* 2).

**Table 2:** Recurrence and cure rates according to patient cohort and intraoperative parathyroid hormone measurement

	IOPTH measurement			No IOPTH measurement			Overall	
	Recurrence rate (%)	Cure rate (%)	No. of patients	Recurrence rate (%)	Cure rate (%)	No of patients	Recurrence rate (%)	Cure rate (%)
Preoperative concordant imaging cohort	2·0	99·5	570	0·2	99·3	2786	0·5	99·3
Heterogeneous cohort	1·3	95·6	1502	0·0	99·2	122	1·7	97·7
Overall	1·5	98·1	2072	0·2	99·3	2908		

IOPTH, intraoperative parathyroid hormone.

An overall complication rate of 4·4 per cent in 4010 patients was reported in nine studies^20^ ^27^, ^28^, ^29^, ^30^, ^31^, ^32^, ^33^, ^34^, whereas five^11^ ^26^, ^35^, ^36^, ^37^ did not report complication rates. Transient and permanent postoperative hypocalcaemia rates were 1·6 and 0·05 per cent respectively. Temporary recurrent laryngeal nerve (RLN) palsy occurred in 1·1 per cent and permanent RLN palsy in 0·3 per cent of the 4010 patients. Complications are summarized in *Table* 3.

**Table 3:** Complications

	No. of complications (*n* = 176)
Temporary RLN palsy seen on laryngoscopy	35 (19·9)
Transient hypocalcaemia defined as < 2 mmol/l	23 (13·1)
Transient hypocalcaemia defined as ≤ 1·95 mmol/l	23 (13·1)
Hypocalcaemia (NOS)	22 (12·5)
Temporary hypoparathyroidism defined as hypocalcaemia (NOS) requiring calcium or vitamin D supplementation, resolving within 6 months	17 (9·7)
Other complication (NOS)	13 (7·4)
Permanent RLN palsy seen on laryngoscopy	11 (6·3)
Wound haematoma requiring return to theatre for evacuation	8 (4·5)
Temporary RLN palsy (laryngoscopy use not documented)	7 (4·0)
Wound infection	4 (2·3)
Myocardial infarction	2 (1·1)
Temporary RLN palsy (selective use of laryngoscopy)	2 (1·1)
Permanent hypoparathyroidism defined as hypocalcaemia (NOS) requiring calcium or vitamin D supplementation, persisting after 6 months	2 (1·1)
Mild neck swelling, managed conservatively	1 (0·6)
Transient hypercalcaemia returning to normal	1 (0·6)
Wound haematoma, managed conservatively	1 (0·6)
Permanent RLN palsy (laryngoscopy use not documented)	1 (0·6)
Cerebrovascular accident	1 (0·6)
Permanent RLN palsy (selective use of laryngoscopy)	1 (0·6)
30‐day mortality not related to surgery	1 (0·6)

Values in parentheses are percentages. RLN, recurrent laryngeal nerve; NOS, not otherwise specified.

There were significant differences in how complications were defined and reported. In terms of RLN palsy, four studies^20^ ^28^, ^31^ ^40^ routinely used laryngoscopy after surgery, one study^29^ used laryngoscopy in patients with postoperative dysphonia, and another study^30^ explicitly stated that laryngoscopy was not used. The remaining publications did not comment on postoperative laryngoscopy. The definition of hypocalcaemia also varied, from a serum calcium level lower than 2 mmol/l^28^ to 1·95 mmol/l or less^30^, whereas other studies did not define cut‐off levels.

## Discussion

This systematic review has indicated that, despite variations in technique, targeted MIP was associated with low long‐term (at least 1 year) recurrence rates of only about 1·5 per cent.

Overall recurrence rates were lowest and cure rates highest in studies that included patients where there was positive, concordant preoperative localization. Interestingly, the analysis suggested that recurrence and cure rates were better when IOPTH measurements were not used. As indicated in *Table* 2, this appeared to reflect use of MIP without routine IOPTH measurement in patients with concordant imaging. IOPTH measurement seemed to be used more frequently in non‐concordant or image‐negative patients.

Two reports^38^ ^39^ relating to MIP were not included in this systematic review as they did not fulfil the inclusion criteria (mean duration of follow‐up not documented). These studies reported a median follow‐up of 9 (range 0–116) months. The first study^38^ investigated whether MIP was associated with a higher recurrence rate than BNE, and the second^39^ considered variables that might predict recurrence in parathyroidectomy for PHPT. In both studies, Kaplan–Meier curves were constructed to determine disease‐free estimates for MIP and BNE. No statistically significant differences were found between MIP and open parathyroidectomy in either study (*P* = 0·55 and *P* = 0·59 respectively).

A meta‐analysis^14^ of studies comparing focused parathyroidectomy with conventional BNE found that recurrence (odds ratio (OR) 1·08, 95 per cent c.i. 0·59 to 2·00; *P* = 0·80) and failure (OR 0·88, 0·58 to 1·34; *P* = 0·56) rates were comparable. Complication rates were significantly lower in the focused parathyroidectomy arm (OR 0·35, 0·15 to 0·84; *P* = 0·02), predominantly related to a lower risk of transient hypocalcaemia (OR 0·36, 0·14 to 0·90; *P* = 0·03). The present review is consistent with these findings, with a 1·6 per cent temporary and 0·05 per cent permanent postoperative hypocalcaemia rate after MIP.

This review also demonstrated the overall complication rate to be low (4·4 per cent in 4010 patients), and similar to values reported by previous large studies^15^ ^40^ involving BNE, together with similar rates of permanent and temporary recurrent laryngeal nerve palsy (0·3 and 1·1 per cent respectively). The lack of postoperative laryngoscopy in the present review and in other studies, however, may mean that the true rate of recurrent nerve palsy is underestimated. Similar consideration applies to rates of postoperative hypocalcaemia, owing to the different cut‐off levels.

This systematic review analysed 14 level 2b studies with a median MINORS score of 10 (mean 9·8), indicating evidence of fair quality. However, its main limitation was the lack of uniformity in the presentation and reporting of data by the individual studies. This is a widely recognized and inherent problem of collating data from observational studies.

Despite these shortcomings, targeted MIP in the surgical management of patients with a presumed single overactive parathyroid adenoma is a safe technique that provides long‐term cure. The review also suggests that MIP is effective without intraoperative hormone estimations in patients with preoperative concordant imaging.

## Disclosure

The authors declare no conflict of interest.

## References

[1] Yeh MW , Ituarte PHG , Zhou HC , Nishimoto S , Liu IL , Harari A *et al* Incidence and prevalence of primary hyperparathyroidism in a racially mixed population. J Clin Endocrinol Metab 2013; 98: 1122–1129. 23418315 10.1210/jc.2012-4022 PMC3590475

[2] Thompson DF . Understanding financial conflicts of interest. N Engl J Med 1993; 329: 573–576. 8336759 10.1056/NEJM199308193290812

[3] Fraser WD . Hyperparathyroidism. Lancet 2009; 374: 145–158. 19595349 10.1016/S0140-6736(09)60507-9

[4] Delbridge LW , Palazzo FF . First parathyroid surgeon: Sir John Bland‐Sutton and the parathyroids. ANZ J Surg 2007; 77: 1058–1061. 17973666 10.1111/j.1445-2197.2007.04324.x

[5] Bellantone R , Raffaelli M , De Crea C , Traini E , Lombardi CP . Minimally‐invasive parathyroid surgery. Acta Otorhinolaryngol Ital 2011; 31: 207–215. 22065831 PMC3203720

[6] Cheung K , Wang TS , Farrokhyar F , Roman SA , Sosa JA . A meta‐analysis of preoperative localization techniques for patients with primary hyperparathyroidism. Ann Surg Oncol 2012; 19: 577–583. 21710322 10.1245/s10434-011-1870-5

[7] Van Husen R , Kim LT . Accuracy of surgeon‐performed ultrasound in parathyroid localization. World J Surg 2004; 28: 1122–1126. 15490067 10.1007/s00268-004-7485-2

[8] Miura D , Wada N , Arici C , Morita E , Duh QY , Clark OH . Does intraoperative quick parathyroid hormone assay improve the results of parathyroidectomy? World J Surg 2002; 26: 926–930. 11965444 10.1007/s00268-002-6620-1

[9] Sebag F , Hubbard JG , Maweja S , Misso C , Tardivet L , Henry JF . Negative preoperative localization studies are highly predictive of multiglandular disease in sporadic primary hyperparathyroidism. Surgery 2003; 134: 1038–1041. 14668738 10.1016/j.surg.2003.07.021

[10] Irvin GL III , Deriso GT III . A new, practical intraoperative parathyroid hormone assay. Am J Surg 1994; 168: 466–468. 7977975 10.1016/s0002-9610(05)80101-1

[11] Kim WW , Rhee Y , Ban EJ , Lee CR , Kang SW , Jeong JJ *et al* Is focused parathyroidectomy appropriate for patients with primary hyperparathyroidism? Ann Surg Treat Res 2016; 91: 97–103. 27617249 10.4174/astr.2016.91.3.97 PMC5016607

[12] Brunaud L , Zarnegar R , Wada N , Ituarte P , Clark OH , Duh QY . Incision length for standard thyroidectomy and parathyroidectomy: when is it minimally invasive? Arch Surg 2003; 138: 1140–1143. 14557134 10.1001/archsurg.138.10.1140

[13] Kunstman JW , Udelsman R . Superiority of minimally invasive parathyroidectomy. Adv Surg 2012; 46: 171–189. 22873039 10.1016/j.yasu.2012.04.004

[14] Jinih M , O'Connell E , O'Leary DP , Liew A , Redmond HP . Focused *versus* bilateral parathyroid exploration for primary hyperparathyroidism: a systematic review and meta‐analysis. Ann Surg Oncol 2017; 24: 1924–1934. 27896505 10.1245/s10434-016-5694-1

[15] Udelsman R , Lin Z , Donovan P . The superiority of minimally invasive parathyroidectomy based on 1650 consecutive patients with primary hyperparathyroidism. Ann Surg 2011; 253: 585–591. 21183844 10.1097/SLA.0b013e318208fed9

[16] Gracie D , Hussain SSM . Use of minimally invasive parathyroidectomy techniques in sporadic primary hyperparathyroidism: systematic review. J Laryngol Otol 2012; 126: 221–227. 22032618 10.1017/S0022215111002908

[17] Westerdahl J , Bergenfelz A . Unilateral *versus* bilateral neck exploration for primary hyperparathyroidism: five year follow‐up of a randomized controlled trial. Ann Surg 2007; 246: 976–980. 18043099 10.1097/SLA.0b013e31815c3ffd

[18] Udelsman R , Donovan PI . Open minimally invasive parathyroid surgery. World J Surg 2004; 28: 1224–1226. 15517494 10.1007/s00268-004-7600-4

[19] Udelsman R . Six hundred fifty‐six consecutive explorations for primary hyperparathyroidism. Ann Surg 2002; 235: 665–670. 11981212 10.1097/00000658-200205000-00008 PMC1422492

[20] Barczynski M , Konturek A , Cichon S , Hubalewska‐Dydejczyk A , Golkowski F , Huszno B . Intraoperative parathyroid hormone assay improves outcomes of minimally invasive parathyroidectomy mainly in patients with a presumed solitary parathyroid adenoma and missing concordance of preoperative imaging. Clin Endocrinol (Oxf) 2007; 66: 878–885. 17437518 10.1111/j.1365-2265.2007.02827.x

[21] National Institute for Health and Care Excellence . *Minimally Invasive Video‐Assisted Parathyroidectomy*; 2014. https://www.nice.org.uk/Guidance/IPG501 [accessed 23 November 2017].

[22] Liberati A , Altman DG , Tetzlaff J , Mulrow C , Gøtzsche PC , Ioannidis JP *et al* The PRISMA statement for reporting systematic reviews and meta‐analyses of studies that evaluate healthcare interventions: explanation and elaboration. BMJ 2009; 339: b2700. 10.1136/bmj.b2700 PMC2714672 19622552

[23] Huang X , Lin J , Demner‐Fushman D . Evaluation of PICO as a knowledge representation for clinical questions. AMIA Annu Symp Proc 2006: 359–363. 17238363 PMC1839740

[24] Slim K , Nini E , Forestier D , Kwiatkowski F , Panis Y , Chipponi J . Methodological Index for Non‐Randomized Studies (MINORS): development and validation of a new instrument. ANZ J Surg 2003; 73: 712–716. 12956787 10.1046/j.1445-2197.2003.02748.x

[25] CEBM. *Oxford Centre for Evidence‐based Medicine – Levels of Evidence (March 2009)* http://www.cebm.net/oxford-centre-evidence-based-medicine-levels-evidence-march-2009/ [accessed 23 November 2017].

[26] Chen H , Pruhs Z , Starling JR , Mack E . Intraoperative parathyroid hormone testing improves cure rates in patients undergoing minimally invasive parathyroidectomy. Surgery 2005; 138: 583–590. 16269285 10.1016/j.surg.2005.06.046

[27] Cohen MS , Finkelstein SE , Brunt LM , Haberfeld E , Kangrga I , Moley JF *et al* Outpatient minimally invasive parathyroidectomy using local/regional anesthesia: a safe and effective operative approach for selected patients. Surgery 2005; 138: 681–689. 16269297 10.1016/j.surg.2005.07.016

[28] Barczyński M , Papier A , Kenig J , Nawrot I . A retrospective case‐controlled study of video‐assisted *versus* open minimally invasive parathyroidectomy. Wideochir Inne Tech Maloinwazyjne 2014; 4: 537–547. 10.5114/wiitm.2014.45087 PMC4280416 25561991

[29] Chow TL , Choi CY , Lam SH . Focused parathyroidectomy without intra‐operative parathyroid hormone monitoring for primary hyperparathyroidism: results in a low‐volume hospital. J Laryngol Otol 2015; 129: 788–794. 26072937 10.1017/S0022215115000651

[30] Yang Z , Guo M , Wu B , Zheng Q , Fan Y . Focused parathyroidectomy through an open‐lateral approach for treating solitary parathyroid adenoma. Surg Pract 2015; 19: 160–165.

[31] Norlén O , Wang KC , Tay YK , Johnson WR , Grodski S , Yeung M *et al* No need to abandon focused parathyroidectomy: a multicenter study of long‐term outcome after surgery for primary hyperparathyroidism. Ann Surg 2015; 261: 991–996. 25565223 10.1097/SLA.0000000000000715

[32] Mihai R , Palazzo FF , Gleeson FV , Sadler GP . Minimally invasive parathyroidectomy without intraoperative parathyroid hormone monitoring in patients with primary hyperparathyroidism. Br J Surg 2007: 94: 42–47. 17083106 10.1002/bjs.5574

[33] Fouquet T , Germain A , Zarnegar R , Klein M , De Talance N , Claude Mayer J *et al* Totally endoscopic lateral parathyroidectomy: prospective evaluation of 200 patients. ESES 2010 Vienna presentation. Langenbecks Arch Surg 2010: 395: 935–940. 20694475 10.1007/s00423-010-0687-1

[34] Venkat R , Kouniavsky G , Tufano RP , Schneider EB , Dackiw AP , Zeiger MA . Long‐term outcome in patients with primary hyperparathyroidism who underwent minimally invasive parathyroidectomy. World J Surg 2012: 36: 55–60. 22089919 10.1007/s00268-011-1344-8

[35] Day KM , Elsayed M , Monchik JM . No need to abandon focused unilateral exploration for primary hyperparathyroidism with intraoperative monitoring of intact parathyroid hormone. J Am Coll Surg 2015; 221: 518–523. 26122588 10.1016/j.jamcollsurg.2015.04.013

[36] Thier M , Nordenström E , Almquist M , Bergenfelz A . Results of a fifteen‐year follow‐up program in patients operated with unilateral neck exploration for primary hyperparathyroidism. World J Surg 2016; 40: 582–588. 26661636 10.1007/s00268-015-3360-6

[37] Nijhuis A , Kluijfhout W , van Dalen T , Twigt BA . Long‐term hypercalcaemia recurrence risk following successful minimally invasive parathyroidectomy. Ann Surg Oncol 2015; 22(Suppl 1): S98–S99.

[38] Schneider DF , Mazeh H , Sippel RS , Chen H . Is minimally invasive parathyroidectomy associated with higher recurrence compared to bilateral exploration? Analysis of over 1000 cases. Surgery 2012; 152: 1008–1015. 23063313 10.1016/j.surg.2012.08.022 PMC3501613

[39] Schneider DF , Mazeh H , Chen H , Sippel RS . Predictors of recurrence in primary hyperparathyroidism: an analysis of 1386 cases. Ann Surg 2014; 259: 563–568. 24263316 10.1097/SLA.0000000000000207 PMC4250051

[40] Allendorf J , DiGorgi M , Spanknebel K , Inabnet W , Chabot J , Logerfo P . 1112 consecutive bilateral neck explorations for primary hyperparathyroidism. World J Surg 2007; 31: 2075–2080. 17768656 10.1007/s00268-007-9068-5

